# EML4‐ALK biology and drug resistance in non‐small cell lung cancer: a new phase of discoveries

**DOI:** 10.1002/1878-0261.13446

**Published:** 2023-05-15

**Authors:** Mariam Elshatlawy, Josephina Sampson, Katy Clarke, Richard Bayliss

**Affiliations:** ^1^ Faculty of Biological Sciences, School of Molecular and Cellular Biology University of Leeds UK; ^2^ Astbury Centre for Structural Molecular Biology University of Leeds UK; ^3^ Leeds Cancer Center, St.James' University Hospital Leeds Teaching Hospitals NHS Trust UK

**Keywords:** cancer, drug resistance, NSCLC, signalling, tyrosine kinase inhibitors

## Abstract

Anaplastic lymphoma kinase (ALK) can be driven to oncogenic activity by different types of mutational events such as point‐mutations, for example F1174L in neuroblastoma, and gene fusions, for example with echinoderm microtubule‐associated protein‐like 4 (EML4) in non‐small cell lung cancer (NSCLC). EML4‐ALK variants result from different breakpoints, generating fusions of different sizes and properties. The most common variants (Variant 1 and Variant 3) form cellular compartments with distinct physical properties. The presence of a partial, probably misfolded beta‐propeller domain in variant 1 confers solid‐like properties to the compartments it forms, greater dependence on Hsp90 for protein stability and higher cell sensitivity to ALK tyrosine kinase inhibitors (TKIs). These differences translate to the clinic because variant 3, on average, worsens patient prognosis and increases metastatic risk. Latest generation ALK‐TKIs are beneficial for most patients with EML4‐ALK fusions. However, resistance to ALK inhibitors can occur via point‐mutations within the kinase domain of the EML4‐ALK fusion, for example G1202R, reducing inhibitor effectiveness. Here, we discuss the biology of EML4‐ALK variants, their impact on treatment response, ALK‐TKI drug resistance mechanisms and potential combination therapies.

## Graphical abstract

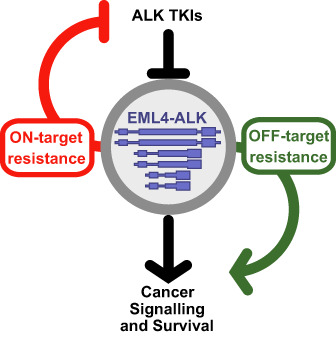

**Abbreviations**
ALK: anaplastic lymphoma kinase
EML4: echinoderm microtubule associated protein like 4
FDA: US Food and Drug Administration
NSCLC: non‐small cell lung cancer
OS: overall survival
PFS: progression‐free survival
TAPE: tandem atypical propeller EML
TD: trimerisation domain
TKD: tyrosine kinase domain
TKI: tyrosine kinase inhibitor
WHO: World Health Organization

## 1 Introduction

### 1.1 EML4‐ALK in non‐mall cell lung cancer

Lung cancer is one of the most common cancers and the primary cause of cancer death globally. According to statistical data by the World Health Organization (WHO), 2.21 million new cases of lung cancer were diagnosed in 2020, of which 81.4% cases were fatal [1]. About 80–85% of all lung cancer cases occur in a prominent type of epithelial lung cancer known as non‐small cell lung cancer (NSCLC) [2]. Histopathological testing reveals three broad subtypes of NSCLC: (a) squamous cell carcinoma, (b) non‐small cell carcinoma and (c) adenocarcinoma [3]. Between 10% and 20% of lung cancer cases are in people who have never (or rarely) smoked. Adenocarcinoma NSCLC is the most common type of lung cancer to be detected in nonsmokers compared with smokers [4, 5]. Progression‐free survival (PFS) and overall survival (OS) rates for NSCLC are improving. As data gathered by National Cancer Institute in the years 2001–2018 show the probability of achieving a three‐year survival rate has risen by over 21% and 31% in NSCLC patients with localised and nonlocalised (i.e. spread or nonspread) disease, respectively [6]. This is collectively due to the therapeutic development of tyrosine kinase inhibitors (TKIs) and improvements in diagnosis. Perhaps the most striking recent improvements in lung cancer survival are seen among patients whose cancers are driven by a distinctive molecular event, the fusion between echinoderm microtubule‐associated protein‐like 4 (EML4) and anaplastic lymphoma kinase (ALK). The EML4‐ALK oncogene is a driver event in 5% of all NSCLC cases and was discovered in 2007 [7, 8].

### 1.2 EML4‐ALK fusion variants

**Fig. 1:**
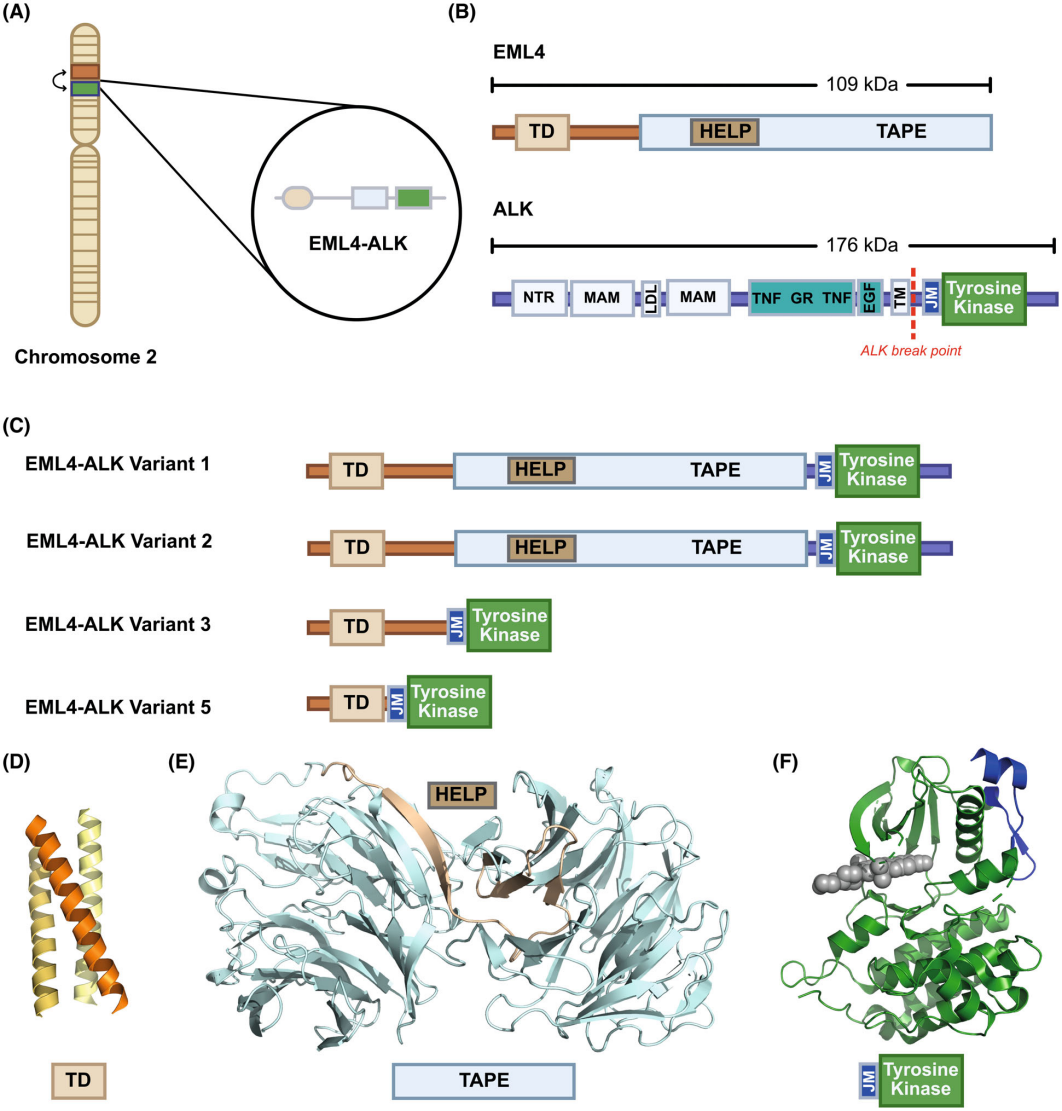
Protein domain structures of EML4, ALK and EML4‐ALK fusion variants. (A) The EML4‐ALK fusion oncogene in NSCLC is the result of an inversion between EML4 and ALK genes on the short *p* arm of chromosome 2, at 2p23.2. (B) The domain structures of EML4 and ALK proteins, of which the trimerisation domain (TD), the juxtamembrane region (JM) and the tyrosine kinase domain are common to all EML4‐ALK fusions. (C) The most frequent EML4‐ALK variants found in NSCLC. (D–F) Crystal structures of: (D) EML4 TD – PDB:4CGC; (E) EML1 TAPE – PDB:4CI8; (F) ALK JM and tyrosine kinase – PDB:3AOX.

Anaplastic lymphoma kinase is a member of the receptor tyrosine kinase family known for regulating cell growth and is activated through binding of the extracellular ligands ALKAL1 and ALKAL2 (also known as FAM150A and FAM150B) [9, 10, 11]. ALK consists of 1620 amino acids and comprises a large, extracellular domain that recognises ligands and a smaller tyrosine kinase domain (TKD) that transmits mitogenic signals through MAPK and other signalling pathways. EML4 is a microtubule‐associated protein that contributes to chromosome congression during mitosis [12]. Consisting of 981 amino acids, EML4 comprises an N‐terminal trimerisation domain (TD), a basic linker region that binds microtubules, and a C‐terminal tandem atypical propeller EML (TAPE) domain [13, 14]. Variants of EML4‐ALK variants result from different breakpoints, giving rise to fusion proteins of different sizes. The ALK gene breaking point mostly occurs at exon 20, with rare examples at exon 19, while the breaking point for EML4 can vary [15]. Hence, different forms of EML4‐ALK fusion proteins are generated including the most common variants, Variant 1 and Variant 3 (Fig. 1) (Table 2). All EML4‐ALK variants include the TD of EML4 and the TKD of ALK, and indeed Variant 5 is a minimal unit with ligand‐independent kinase activity that drives proliferation. Variant 3 includes the basic region and shows strong microtubule localisation, especially when the kinase is inhibited or inactivated by mutation [16]. Other variants are longer and, strikingly, include only a partial TAPE domain that must therefore be misfolded. Indeed, the longer variants exhibit increased dependence on molecular chaperones and are highly sensitive to Hsp90 inhibition [14].

The relative occurrence of the variants detected in studies is variable due to factors such as patient sample size, the molecular screening tool used to identify variants and the ethnicity of participants. For example, a study by Lin and collaborators included 129 NSCLC participants with an ALK alteration (ALK‐positive or ALK+) and a further 577 patients from a Foundation Medicine dataset showed Variant 1 as most frequently detected at 43% compared to 40% with Variant 3 [17]. Similar results were obtained from another study [18]. However, a group led by Wen on the genomic impact of oncogenes in NSCLC of 1200 Chinese patients yielded an opposite conclusion, consistent with Christopoulos et al., whereby Variant 3 was found most common [19, 20]. Variant 1 was also most common in the global ALEX III trial, at 37.0% and 42.7% in plasma and tissue samples, respectively, while Variant 3 was present at 36.3%/37.1% (plasma/tissue) [21]. These results may suggest heterogeneity in the variant type within some patients, as the variant detected in plasma versus tissue was different in 20% of patients for whom matched samples were analysed. The previous standard methods for detecting the presence of the EML4‐ALK fusion were fluorescence *in situ* hybridisation and immunohistochemistry, which cannot distinguish between variants, and so this information was missing for these patients. However, increasingly, next‐generation sequencing (NGS) is becoming the prevailing method. The use of NGS can not only screen for all four routinely treatable oncogenic fusions (ALK, ROS1, NTRK and RET) but also identify the specific variant present in each sample [22]. The availability of these data will enable robust evaluation of the impact of variant type on each patient's response to therapy.

### 1.3 ALK tyrosine kinase inhibitors (TKIs)

Anaplastic lymphoma kinase‐tyrosine kinase inhibitors currently approved for use in EML4‐ALK+ NSCLC include crizotinib (first generation), ceritinib, alectinib and brigatinib (second generation) and lastly, lorlatinib (third generation; Table 1). Moreover, lorlatinib is approved for use by both the US Food and Drug Administration (FDA) and European Medicines Agency as first‐line therapy and for subsequent lines of treatment, and ensartinib is approved for clinical use in China. With an OS rate of about 75% and a response rate roughly above 60%, crizotinib was approved in 2011 by the FDA as a TKI that targets not only ALK, but also MET and ROS1 proteins [23, 24]. However, ceritinib and alectinib surpassed the potency of crizotinib as they presented higher efficiency against central nervous system (CNS) metastases [25, 26, 27]. Unapproved TKIs such as TPX‐0131 and TQ‐B3139 are still under evaluation and recruitment is ongoing for a Phase III clinical study (NCT04009317) to assess TQ‐B3139 vs. crizotinib as first‐line treatment in EML4‐ALK+ NSCLC [28] (Table 1). Combining drugs that target different pathways can yield better results than using a single drug or drugs that function similarly [29].

**Table 1:** ALK‐TKIs used in the treatment of EML4‐ALK+NSCLC. Red—first‐generation ALK‐TKI, Blue—second‐generation ALK‐TKI, Green—third‐generation ALK‐TKI, Black—fourth‐generation ALK‐TKI (currently in clinical trials).

ALK tyrosine kinase inhibitors used in the treatment of EML4‐ALK+ non‐small cell lung cancer (NSCLC)			
ALK inhibitor drug (commercial name)	Chemical scaffold	Drug target(s)	CNS penetrant
Crizotinib [ 77 ] (Xalkori)	Aminopyridine	ALK c‐MET ROS1 [29]	No [77]
Ceritinib [ 78 ] (Zykadia)	Diaminopyrimidine [78]	ALK IGF‐1 InsR ROS1 [29]	Yes [27]
Alectinib [ 79 ] (Alecensa)	Dihydrobenzocarbazole [79]	ALK STAT3 RET [77]	Yes [79]
Brigatinib [ 80 ] (Alunbrig)	Diaminopyrimidine [80]	ALK FLT3 EGFR INSR ABL1 MET IGF1R ERBB2/4 ROS1 [29]	Yes [80]
Ensartinib [81]	Aminopyridazine‐based [81]	ALK ROS1 [29] MET ABL EPHA2 Axl LTK SLK	Yes [81]
Lorlatinib [ 82 ] (Lorbrena/Lorviqua)	Macrocyclic [82]	ALK [82] ROS1 [29]	Yes [83]
TPX‐0131 [84]	Macrocyclic [84]	ALK [84]	Yes [84]
TQ‐B3139 [28]		ALK [28] c‐MET [28]	Yes [28]
NUV‐655/NVL‐655 [85]		ALK [85] ROS [85]	Yes [85]

The rise of a novel mutation ALK L1256F showed resistance to lorlatinib, whereas I1171 mutations showed insensitivity to crizotinib and alectinib, but not ceritinib (Table 3). On the contrary, the novel mutation F1174C showed sensitivity to alectinib, but not to crizotinib nor ceritinib. According to a recent study, gilteritinib was effective in inhibiting ALK L1256F‐mutated tumours, previously found to be insensitive to lorlatinib. However, gilteritinib was not effective in the context of the ALK mutations G1202R and D1203N [30]. As seen in Table 3, unlike alectinib and crizotinib, ceritinib showed efficacy against the gatekeeper mutation L1196M [25, 27]. In 2017, alectinib was approved as a first‐line treatment because it achieved a PFS median value of 34.8 months in untreated EML4‐ALK+ NSCLC patients of the ALEX study [31]. Ultimately, third‐generation lorlatinib was seen to inhibit relatively more ALK mutations than the aforementioned ALK‐TKIs, as represented in Table 3 [32]. To better manage EML4‐ALK+ NSCLC, it is worth considering side effects associated with ALK‐TKIs. Solomon and colleagues reported visual disturbance and decreased sex hormone levels (hypogonadism) in patients using crizotinib [33]. Although side effects such as gastrointestinal adversities were reportedly lower when alectinib was used compared with crizotinib, alectinib overall showed more toxicity compared with other TKIs [26, 34]. The availability of several different TKIs with different properties raises the question of which is the optimal sequence in which to use them, with the weight of evidence suggesting a ‘best‐first’ approach [35].

## 2 Phase separation of EML4‐ALK fusion proteins

In its normal functional state, the ALK protein is located in the plasma membrane; however, in the context of EML4‐ALK, the extracellular and transmembrane regions of ALK are lost and the oncogenic protein is localised in distinct compartments in the cytoplasm [16, 36] (Table 2). Apart from the characterisation of the molecular and structural differences of EML4‐ALK variants, recent studies revealed an unexpected mechanism by which EML4‐ALK proteins can phase separate to function within the cell. We and others have found that EML4‐ALK proteins form cytoplasmic compartments and recruit proteins to orchestrate oncogenic signalling of RAS/MAPK and JAK/STAT pathways [16, 36]. The formation of these higher order protein assemblies depends on the conformational state of the catalytic domain of ALK kinase and the transient association of stable EML4‐ALK trimers. Catalytically inactive mutants do not form compartments and the ALK‐TKIs, ceritinib and lorlatinib, dissolve EML4‐ALK cytoplasmic compartments and redirect Variant 3, but not Variant 1, to microtubules [16]. In contrast, cytoplasmic EML4‐ALK compartments are more evident in constitutively active ALK mutants such as in the presence of the F1174L point mutation and are stabilised by a second‐generation ALK‐TKI, alectinib. Compartment formation is therefore dependent on ALK being active or in a conformational state that mimics the kinase in its active form. The presence of a portion of EML4 in the fusion is also important for the formation of these compartments providing solid evidence that the fusion partner of ALK is essential for its function [7, 37].

**Table 2:** Comparison of EML4‐ALK Variant 1 and Variant 3 variants. NL20, nontumorigenic cell line derived from human bronchial epithelium expressed active phosphorylated EML4‐ALK; PFS, patient‐free survival.

	Comparison between variants	
	Variant 1	Variant 3
Protein stability	Low [14, 86, 87]	High [14, 86]
Protein localisation	Compartment, solid‐like [16, 36]	Compartment, liquid‐like, [16, 36], Microtubule [13, 72]
Omics	H3122 transcriptome [70] H3122 phosphoproteome [80] NL20‐Variant 1 transcriptome & phosphoproteome [70]	H2228 transcriptome [70] NL20‐Variant 3 transcriptome & phosphoproteome [70]
Clinical impact	Longer PFS [20, 42] Low metastasis [20, 40]	Shorter PFS [20, 42] High metastasis [20, 40]

Intriguingly, the importance of these EML4‐ALK compartments in tumorigenesis, and drug tolerance has been highlighted recently [37, 38]. Gonzalez‐Martinez et al. have sought to test the role of oncogenic EML4‐ALK compartments in drug response and resistance development. Interestingly, they found that EML4‐ALK compartments trap signalling molecules such as GRB2 and suppress any other signalling activated by membrane‐associated RTKs. ALK inhibition with crizotinib dissolves EML4‐ALK compartments and, subsequently releases GRB2 protein to relocate to membrane RTKs such as EGFR, allowing RTK signals to be restored. In another study, Qin et al have shown that EML4‐ALK variant 1 can drive tumorigenesis in genetically engineered mouse models and analysis of murine tumours and primary tumour‐derived organoids contained high numbers of EML4‐ALK compartments [37]. Nonetheless, the discovery of these phase‐separated EML4‐ALK compartments opens new avenues in better understanding their cellular functions and contribution to tumorigenesis and drug resistance.

## 3 Clinical differences between variants

One clinical difference between the variants is their propensity to drive metastasis, as described previously [19, 39]. In one study, a higher proportion of patients having shorter variants (i.e. Variant 3 and Variant 5) exhibited metastases at diagnosis (69% vs 47%) than patients having longer variants (i.e. Variant 1 and Variant 2.) [39] (Table 2). In the other study, Variant 3 expression showed an increased frequency in the number of metastases compared with other variants (mean metastatic sites at diagnosis 3.3 vs. 1.9 and 1.6, p = 0.005) [20]. Increased metastasis might be due to the higher motility of cells expressing EML4‐ALK variant 3 [39], via a novel mechanism dependent on cellular protrusions formed by microtubules and their associated kinases NEK7 and NEK9 [40].

Several studies have reported on the question of whether different EML4‐ALK variants affect disease progression and treatment response. These were brought together in a recent scholarly review [41], and so we will cover this topic briefly. Earlier studies, based on data from patients treated (or treated initially) with the first‐generation inhibitor crizotinib, showed a significantly high rate of progression and worse survival for Variant 3 than other variants [20, 39, 42]. However, data from the global Alex III trial that compared alectinib with crizotinib found that the PFS for Variant 3 and Variant 2 patients were numerically worse than for Variant 1 patients, but these were not statistically significant for either inhibitor. Another study reported that Variant 3 patients treated with the third‐generation inhibitor lorlatinib had significantly longer PFS than Variant 1 patients [17], while a second study on lorlatinib found no such difference [43]. These analyses are complicated by other factors that might correlate with variant type—such as the contribution of mutations in other genes. For example, TP53 mutations are present in about 20% of EML4‐ALK patients of all variant types, and patients who have a combination of a TP53 mutation and EML4‐ALK Variant 3 present with significantly more metastases and have a worse prognosis [44]. Overall, studies suggest that the type of the EML4‐ALK fusion protein confers differential response and resistance to ALK‐TKIs (Table 2).

## 4 Drug‐resistance mechanisms

### 4.1 On‐target drug‐resistance mechanisms in EML4‐ALK+NSCLC

**Fig. 2:**
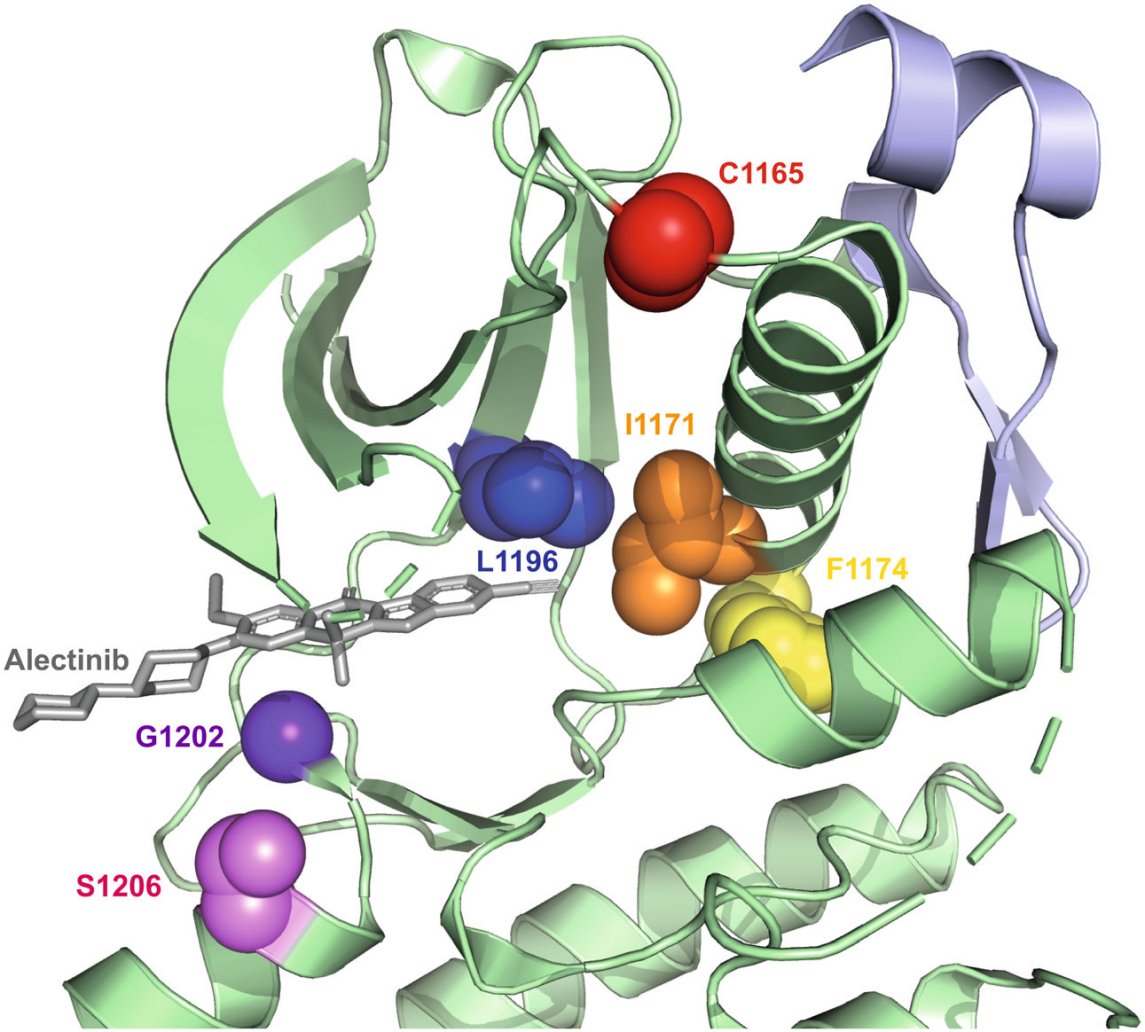
Structure of ALK kinase domain and key mutation sites. View of ALK kinase domain (green) bound to alectinib (grey) centred on the active site highlighting the key residues that are frequently mutated mapped onto the crystal structure (PDB:3AOX). Red: C1156 residue, Orange: I1171 residue, Yellow: F1174 residue, Blue: L1196 residue, Indigo: G1202 residue, Violet: S1206 residue. The juxtamembrane region of ALK is shown in light purple.

Resistance to treatment can be due to secondary mutations such as gene amplification or mutations in the ALK TKD (Table 4). These mutations can result in enhanced kinase activity and/or reduced binding of TKIs [45, 46]. As seen in Table 3, on‐target resistance mechanisms include prominent gatekeeper and solvent‐front mutations such as L1196M and G1202R [46, 47]. The L1196M mutation in EML4‐ALK is in the gatekeeper residue at the back of the ATP‐binding pocket (Fig. 2), and is analogous to the EGFR‐based T790M mutation that is resistant to TKIs such as gefitinib [45].

The G1202R mutation is in the solvent‐exposed binding site at the front of the ATP‐binding site (Fig. 2), in a prime position to disrupt the binding of TKIs that exploit this region of the kinase structure to enhance potency, selectivity and solubility. Indeed, this change is analogous to the difference in sequence at the equivalent position of PLK1 (R136) and NEK2 (G92) that was used in the development of selective NEK2 inhibitors [48]. G1202R is a much more common resistance mutation in Variant 3 than Variant 1—in one study, 32% of patients harbouring Variant 3 expressed the G1202R mutation, which was absent in all patients of Variant 1—and consistent results were found in other studies [17, 20, 41, 49]. Mutations at other positions in the ATP‐binding pocket, such as G1269A that is one residue N‐terminus to the kinase DFG‐motif, also builds resistance to crizotinib by causing steric hindrance upon inhibitor binding [45]. In contrast, mutations such as C1156Y and F1174C act through allosteric activation of the kinase (Fig. 2) [50].

The rise of compound mutations further complicates the analysis of drug resistance mechanisms. Exposure of lung epithelial cells expressing EML4‐ALK with single resistance mutations to lorlatinib resulted in multiple clones with different compound mutations, including L1196M or L1198F arising from G1202R model cells [51]. EML4‐ALK lung cancer patients do not necessarily express only one secondary mutation but can express double and even triple mutations such as S1206F/G1202R/G1269A simultaneously as a scheme of increasing on‐target resistance [32]. For instance, Zhu and collaborators identified a novel variant 3 G1202R/S1206Y double mutation in cis in a patient that was sequentially treated with crizotinib, alectinib and then lorlatinib ([32]; Table 3). Other resistance mutations in ALK have also been discovered such as [p.A1200_G1201delinsW] in exon 23 of ALK, which alters the shape of the solvent‐exposed edge of the ATP‐binding pocket [52]. Several studies correlated heterogeneous resistance to ALK‐TKI treatment concluding the importance of gene profiling in detecting mutations and variants for effective clinical management of EML4‐ALK+ NSCLC [18, 52].

**Table 3:** Drug‐Resistant mutations after ALK‐TKIs treatment. (?) means the sensitivity of this mutation was not assessed against the given drug; (active) means mutation in that row is inhibited by the addressed drug. The differential colour coding highlights the key residues on the ALK kinase domain, as seen in Fig. 2.

EML4‐ALK kinase inhibitors & drug‐resistant mutations				
Crizotinib	Ceritinib	Alectinib	Brigatinib	Lorlatinib
L1196M [46]	?	L1196M	?	?
G1269A [88]	?	?	?	?
C1156Y [45]	C1156Y/T	?	?	?
I1171T/N/S	Active	I1171T/N/S	?	?
S1206 C/Y [ 64 ]	Active	Active	S1206C	?
E1210K [45]	Active	Active	E1210K	?
L1152P/R [46]	L1152P/R	?	?	?
V1180L [88]	Active	V1180L	?	?
I1151T [46, 88]	I1151T	?	?	?
F1174C [89]	F1174C/L	?	?	?
F1245C [90]	Active	?	?	?
G1202R [ 56 ]	G1202R	G1202R	G1202R	?
E1210K [53]	Active	Active	E1210K	?
D1203N [53]	Active	Active	D1203N	?
Active	?	?	?	L1198F [91]
?	?	Active	?	L1256F [92]
?	?	?	?	G1202R/S1206Y [32]

### 4.2 Off‐target resistance mechanisms in EML4‐ALK+NSCLC

Off‐target resistance mechanisms occur in many cases in response to ALK‐TKIs [53, 54, 55] (Table 4). These involve the upregulation or activation of alternative tyrosine kinase receptors including, but not limited to, EGFR SRC, and KIT that ultimately trigger their respective bypass pathways [46, 56]. EML4‐ALK protein is involved in complex molecular interactions across multiple downstream pathways such as EGFR, MEK/ERK, JAK/STAT, PI3K/AKT, KIT and HER family signalling. As such, dysregulation in the function of this collaborative network can lead to acquired resistance [57]. Activation of the HER family pathway can cause acquired resistance in patients who receive ALK‐TKI treatment [57]. Crizotinib‐resistant cell lines derived from EML4‐ALK+ lung cancer showed elevated levels of EGFR, HER2 and HER3 phosphorylation [57]. Ceritinib‐resistant (H3122‐CER) and TAE684‐resistant H3122 cell lines acquired resistance through EGFR bypass pathway activation [58, 59]. Resistance can also be mediated through RAS mutations, such as Q61K [52].

**Table 4:** Resistance mechanisms in EML4‐ALK+positive NSCLC patients.

Types of resistance mechanisms		
On‐target resistance	Off‐target resistance	Additional resistance
ATP site Gatekeeper mutations [64]	KRAS‐exon 3 mutation [52]	Anti‐apoptotic pathways [93]
ATP site solvent‐front mutations (e.g.: G1202R) [64]	MET amplification [64]	Histological transformation [94]
Second‐site mutations [46, 95]	MEK activation [96]	Epigenetics [97]
Compound mutations	PIK3CA mutations [98]	Tumour microenvironment [29]
Oncogene amplification [46]	IGF‐1R activation [56]	
Oncogene loss [46]	KRAS G12C mutation [46]	
ALK‐exon 23 mutation [52]	RET fusion [52]	
	MAPK pathway [68]	
	TP53 mutation [44]	
	PI3K‐AKT pathway [99]	
	JAK–STAT pathway [60]	
	SRC activation [100]	
	EGFR activation [58]	
	KIT activation [64]	
	HER family activation [58] YAP activation [62, 66]	

An example showing overlap of on‐ and off‐target resistance mechanisms working together can be seen with G1202R mutation and the Slug pathway. By activating the Slug signalling pathway and concomitantly upregulating STAT3, the G1202R mutation develops resistance against ceritinib by inducing an EMT phenotype (Epithelial–Mesenchymal Transition) that significantly increases cell migration [60]. A combination of ALK and STAT3 inhibitors managed to restore the sensitivity of G1202R mutant cells to ceritinib [60]. Hypoxia can also cause resistance to ALK‐TKIs through induction of EMT [29, 61]. This mechanism could be targeted through dual inhibition of SRC and ALK [55].

Another example of off‐target resistance is TP53 mutation, whereby a poor PFS can be correlated to TP53 exclusively treated with crizotinib [62]. EML4‐ALK Variant 3 was found in 30–40% of a patient cohort study run by Christopoulos et al. exhibiting TP53 mutation, although Variant 3 and TP53 mutations exist independently of one another [19]. TP53 mutation was associated with a significantly shorter PFS of only 8 months in the multi‐ALK‐TKI group compared with wild‐type TP53 in the crizotinib‐only group with a PFS of 13 months. Moreover, finding a way to target TP53 mutations is a priority as they are commonly observed in genomic co‐alterations that contribute to heterogeneous responses in EML4‐ALK+ NSCLC [41]. Interestingly, several studies have shown that after the sequential use of multiple ALK‐TKI treatments, concomitant ALK‐activating mutations and activation of bypass signalling pathways are more likely to occur. For example, the dual activation of bypass signalling pathways in the multiple ALK‐TKI group was 29% compared to 6% in the crizotinib‐only treatment group [62].

## 5 Combination therapy

Using ALK‐TKIs as a single treatment for patients with ALK‐rearranged NSCLC is probably not the long‐term answer for optimal clinical activity and the search is on for combinations that prevent or overcome resistance. The genetics of NSCLC is a complex landscape of driver oncogene mutations working together with further mutation, providing additional targets that could be the basis of rational combinations. One study calculated that co‐alterations of EGFR and ALK were calculated at a frequency of 5.01% [63]. Activation of EGFR is a resistance mechanism purposefully adopted by EML4‐ALK cells to evade cell death; therefore, EGFR‐inhibitor drugs erlotinib or afatinib were used in combination with crizotinib to inhibit H3122 cells [24]. In addition, erlotinib or afatinib in combination with ALK‐TKI yielded robust results compared with imatinib, an inhibitor of KIT, ABL and PDGFR [64]. Assessing the use of cetuximab was also attempted; however, there have been conflicts associated with its use in cell line cultures as it is a monoclonal antibody for EGFR [24]. The transcriptional activation of Yes‐associated proteins (YAP) is elevated in pre‐ and post‐treated sample/cells [65, 66]. Increased expression of YAP is associated with poor response to ALK inhibitors and survival of EML4‐ALK+ NSCLC cells. The combinatorial therapy of YAP1 with ALK inhibitors showed tumour remission in ALK‐rearranged xenografts [66]. There is the potential for other combinations of oncogenes because 73.9% of NSCLC patients expressed a minimum of one gene alteration in genes such as EGFR, ALK, ERBB2, MET, BRAF, ROS1 and RET [19]. Further analysis is needed to calculate what proportion of patients have double hit mutations in ALK and another oncogene such as EGFR, and whether this is adequately provided for in current diagnosis and treatment. In another study, inhibition of CDK7/12 with THZ1 and CDK9 with alvocidib or dinaciclib were remarkably effective in parental and resistant‐ crizotinib, ceritinib or alectinib EML4‐ALK cells [67]. Targeting the key signalling pathways downstream of EML4‐ALK is another strategy to be explored in the optimisation of therapy. For example, RAS‐MAPK is critical for the survival of EML4‐ALK variant 1 cells, and a combination of the MEK inhibitor trametinib enhanced response and delayed resistance to crizotinib and ceritinib in relevant *in vitro* and *in vivo* models [68]. In fact, a phase 1 clinical study is currently investigating the combination of an ALK‐TKI, ceritinib, with a MEK inhibitor, trametinib, in ALK+ or ROS1+ NSCLC patients [69]. A study by Chuang et al. highlighted the importance of SERPINB4, a protease inhibitor protein, in EML4‐ALK and other ALK fusions. Cell lines expressing ALK fusions evade cell death by inhibiting NF‐kB and STAT3 pathways via SERPINB4. Targeting these pathways might therefore improve patient response to ALK‐TKIs in NSCLC [70].

EML4‐ALK Variant 3, but not Variant 1, is associated with microtubule stability [13] and therefore the use of microtubule poisons as a single or combination treatment with ALK‐TKIs has been assessed [71, 72]. Traditional chemotherapeutic agents, vincristine and paclitaxel were assessed for their use as single or in combination therapy with ALK inhibitors to sensitise EML4‐ALK cell lines expressing Variant 1 or Variant 3 [71]. The combination of vincristine with ALK‐TKIs, crizotinib or ceritinib, elicited antiproliferative activities and inhibition of signalling pathways in Variant 1, but not in Variant 3‐bearing EML4‐ALK+ NSCLC cells [71]. It was hypothesised that EML4‐ALK Variant 3 cells had low response due to high levels of acetylated tubulin and thus increased microtubule stability. In another study, the use of paclitaxel, a microtubule stabilising drug, in combination with ALK‐TKIs had a synergistic effect in both EML4‐ALK Variant 1 and Variant 3 cells [72]. Both studies highlight the importance of better understanding the differences between EML4‐ALK variants when selecting a combination treatment. An excellent detailed review previously published by Papageorgiou et al highlights the various monotherapy and polytherapy approaches that may be beneficial to specific EML4‐ALK variants [73].

## 6 Challenges and perspectives

**Fig. 3:**
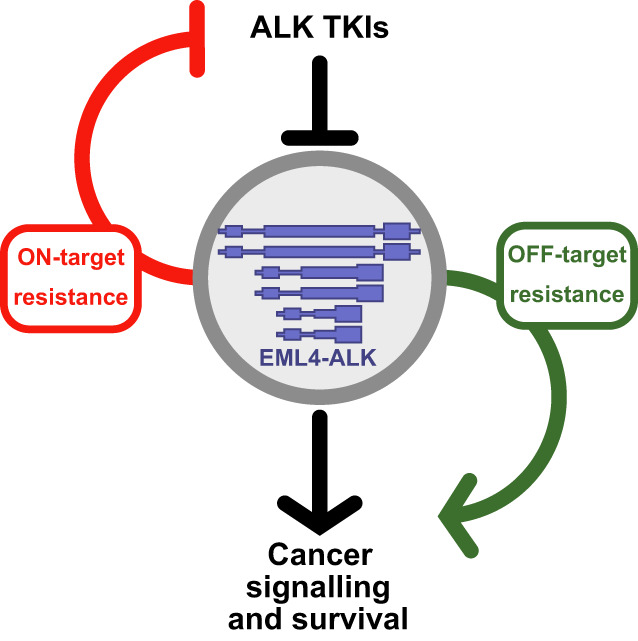
Schematic overview of EML4‐ALK resistance mechanisms. EML4‐ALK variants generate proteins of different sizes that form cellular compartments. This drives cancer signalling and promotes cancer cell survival pathways that can be blocked with ALK‐TKIs. Resistance pathways can be on‐target and diminish the efficacy of ALK‐TKIs; or off‐target, and bypass the requirement for ALK activity altogether.

Drug resistance is an ongoing challenge in NSCLC with not only ALK targeted therapies but also EGFR, ROS1 and other oncogenes. Although ALK‐TKIs are effective and beneficial for the majority of EML4‐ALK+ NSCLC patients, there are patients who are more inhibitor‐insensitive, and there is a need for in‐depth research into the mechanisms that underpin innate drug resistance (Fig. 3). This highlights the urgent need for more studies to achieve this aim and maximise data gathered on mutations and drug sensitivities. As a starting point, testing for EML4‐ALK variants and additional mutations via DNA sequencing is an important analysis that needs to be considered when diagnosing NSCLC patients, albeit at some financial cost, to achieve an effective and personalised therapeutic scheme. This will help address heterogenous prognoses presented to patients that can be due to secondary mutations, resistance mechanisms, or most importantly a result of intratumour genomic heterogeneity [74]. When factors such as additional ALK mutations, TP53 variants, and other ALK variants are better understood, oncologists will be able to select an optimal therapy based on rational criteria. By improving patient response to treatment, the chances of developing a resistance mechanism over a relatively longer time frame than currently witnessed can potentially be decreased [20].

Another priority is to develop an inexpensive yet accurate molecular diagnostic tool for EML4‐ALK+ NSCLC. This would enable early detection of disease, monitoring of patients and could reduce the strain on histopathological laboratories that are having to carry out ever‐increasing analyses across different cancer types. Ideally, the diagnostic tool would include the detection of variants and other key genetic factors. This would facilitate research as well as diagnosis because, to date, research studies and clinical practices use different techniques for diagnosis which complicates analysis and reduces the accuracy of data collected.

Considering resistance by persistent mutations such as G1202R and I1151Tins, and the possibility of compound mutations, there is a debate as to how much further optimisation of ALK‐TKIs is possible, or whether investment in alternative targeting approaches is needed. Because EML4‐ALK fusions lack extracellular domains, approaches based on ALK‐directed antibodies are not feasible. Fortunately, considerable effort is going towards a wider range of approaches, including gene targeting [73, 75]. For example, a recent paper used a gold nanoshell‐based system to deliver microRNA‐301 gene, and multiple ALK siRNAs and chemotherapy drugs [76]. Targeting ALK simultaneously via several modalities might reduce the risk of therapeutic resistance, although the road to routine clinical application is likely to be long and challenging. Nevertheless, we are confident that further improvements in patient outcomes can be achieved through investment in the areas of (a) molecular diagnostic tools, (b) variant and mutation testing, (c) therapeutic modality development, all of which will be required to optimise management of EML4‐ALK+ NSCLC.

## Conflict of interest

The authors declare no conflict of interest.

## Author contributions

ME, JS, KC and RB were involved in conception and design. ME, JS and RB were involved in writing, review and/or revision of the manuscript. All authors have read and agreed to the published version of the manuscript.

## Data Availability

No primary data sets have been generated or deposited.
